# A Comprehensive Analysis of Therapeutic Potential of Medicinal Plant Extracts to Treat Ethanol-Induced Gastric Ulcer

**DOI:** 10.3390/biomedicines14030562

**Published:** 2026-02-28

**Authors:** Raja Singh Paulraj, Anbazhagan Sathiyaseelan, Parthasarathi Perumal, Arunkumar Ramachandran, Shanthi Grace Paulraj

**Affiliations:** 1 Robert C. Byrd Biotechnology Science Center, Marshall University, Huntington, WV 25701, USA; 2 Department of Bio-Health Convergence, Kangwon National University, Chuncheon 24341, Republic of Korea; sathiyaseelan.bio@gmail.com; 3 Department of Molecular and Cell Biology, Greensmed Labs, Chennai 600097, Tamil Nadu, India; drsarathibio@gmail.com; 4 Multidisciplinary Research Unit (A Unit of DHR/ICMR, Ministry of Health and Family Welfare, New Delhi), Madras Medical College, Chennai 600003, Tamil Nadu, India; arunkumarunom@gmail.com; 5 College of Nursing, Government Mohan Kumaramangalam Medical College Hospital, Salem 636001, Tamil Nadu, India; shanthigrace1980@gmail.com

**Keywords:** gastric ulcers, alcohol, inflammation, phytomedicine

## Abstract

**Background/Objectives:** Gastric ulcer is a prevalent global gastrointestinal disorder influenced by multiple factors, including excessive alcohol consumption, poor dietary habits, psychological stress, smoking, and the chronic use of non-steroidal anti-inflammatory drugs. Among these, alcohol plays a critical role in gastric mucosal injury by enhancing gastric acid secretion, triggering inflammatory responses, inducing oxidative stress, and promoting epithelial cell apoptosis while simultaneously depleting key protective mediators such as nitric oxide and prostaglandin E_2_. Growing interest has focused on medicinal plants as promising sources of novel therapeutic agents for the management of peptic ulcer disease. **Methods:** This review summarizes commonly used medicinal plants documented in both Ayurvedic and modern medical systems that exhibit ulcer-healing potential. Experimental and preclinical studies indicate that various herbal drugs and plant extracts derived from different plant parts exert significant anti-ulcer effects through multiple mechanisms, including antioxidant activity, modulation of inflammatory pathways, enhancement of mucosal defense, and inhibition of gastric acid secretion. **Results:** The review further highlights the gastroprotective effects of these herbal remedies as demonstrated in established experimental ulcer models. **Conclusions:** Exploring plant-based therapies for gastric ulcers offers valuable insights into alternative and complementary treatment strategies. Continued research aimed at identifying bioactive compounds, elucidating their molecular mechanisms, and developing improved formulations may contribute to safer, more effective, and patient-friendly therapeutic options for peptic ulcer management.

## 1. Introduction

Peptic ulcer (PU) has long been a major cause of gastrointestinal surgery, with high morbidity and mortality, and has been referred to as the “new plague of the 21st century”. Since 1990, peptic ulcer has accounted for 4404 deaths globally. However, age-standardized rates of prevalence, incidence, and mortality have declined significantly. Autoregressive integrated moving average (ARIMA) models predict that, despite an initial rise in cases, the overall burden will continue to decrease by 2050, reflecting improvements in management and preventive strategies [1,2,3].

PUs are mucosal lesions that extend beyond the muscularis mucosae, forming a cavity encircled by inflammation. They are primarily categorized into gastric ulcers and duodenal ulcers based on their location [2]. PU is a prevalent gastrointestinal illness, affecting a substantial proportion of individuals worldwide, with higher prevalence reported in developing countries. Major contributing factors include the misuse of non-steroidal anti-inflammatory drugs (NSAIDs) and infections with *Helicobacter pylori* (*H. pylori*) [4,5,6,7]. Additional risk factors comprise physical and psychological stress, environmental pollutants, pesticides, heavy metals, and tobacco and alcohol use [8,9,10,11].

The development of a gastric ulcer is multifactorial, resulting from an imbalance between aggressive factors—such as gastric acid, pepsin, ethanol exposure, NSAIDs, and *H. pylori*—and mucosal defensive mechanisms [3,4,12,13,14]. Protective mechanisms include mucus and bicarbonate secretion, prostaglandin synthesis, adequate mucosal blood flow, and antioxidant systems. These factors ultimately converge through shared pathophysiological pathways involving oxidative stress, inflammatory signaling, and disruption of the epithelial barrier [3,12,15].

Current pharmacological management of PU primarily includes proton pump inhibitors, H2 receptor antagonists, antacids, and antibiotic regimens for *H. pylori* eradication. Although these therapies are effective, long-term use is associated with adverse effects, recurrence, drug resistance, and incomplete mucosal healing in some patients. These limitations have stimulated increasing interest in alternative and complementary therapeutic approaches, particularly plant-derived bioactive compounds with antioxidant, anti-inflammatory, and cytoprotective properties [3,12,13,16,17].

Experimental models play a critical role in understanding gastric ulcer pathogenesis and screening potential therapeutic agents. Among these, the ethanol-induced gastric ulcer model is widely employed due to its reproducibility and ability to rapidly induce mucosal damage through oxidative stress, inflammation, lipid peroxidation, and epithelial necrosis. This model closely mimics acute gastric mucosal injury and allows systematic evaluation of gastroprotective mechanisms of medicinal plant extracts [15,18,19,20,21].

Although numerous studies have reported the gastroprotective effects of individual medicinal plants in ethanol-induced ulcer models, the available evidence remains fragmented and mechanistically dispersed across experimental studies. A comprehensive synthesis focusing specifically on the molecular pathways, phytochemical classes, and translational relevance of plant extracts evaluated in ethanol-induced gastric ulcer models is currently lacking. Understanding these mechanisms not only clarifies the actions of bioactive compounds but also guides future translational research in anti-ulcer therapy [15,18,21].

Therefore, this review aims to critically summarize and integrate recent experimental findings on medicinal plant extracts tested in ethanol-induced gastric ulcer models, with particular emphasis on their antioxidant, anti-inflammatory, and cytoprotective mechanisms.

## 2. Selection of Medicinal Plant Extracts

Although this review is narrative in nature, a structured and transparent approach was adopted to identify relevant medicinal plant extracts for inclusion. A comprehensive literature search was conducted using the PubMed, Scopus, and Google Scholar databases to identify experimental studies published between 2001 and 2025 that evaluated plant-derived extracts in ethanol-induced gastric ulcer models. The search strategy included combinations of the following keywords: (“ethanol-induced gastric ulcer” OR “alcohol-induced gastric injury”) and (“medicinal plant” OR “plant extract” OR “phytotherapy” OR “natural compound”). Only articles published in English were considered.

Studies were included if they (i) employed in vivo ethanol-induced gastric ulcer models, (ii) investigated plant-derived extracts or isolated phytochemicals, and (iii) reported quantitative outcomes such as ulcer index, histopathological findings, or mechanistic biomarkers (e.g., antioxidant or inflammatory parameters). Exclusion criteria encompassed review articles, clinical-only studies, studies without clearly described experimental design, or those not directly relevant to ethanol-induced gastric injury.

To enhance transparency and minimize bias, data extraction and standardization were conducted systematically, with doses expressed in mg/kg body weight, ethanol concentrations in % (*v*/*v*), and exposure times in a uniform format. This approach ensured consistency across studies while maintaining the narrative scope of the review.

## 3. Methodological Limitations

While a structured and transparent approach was applied, several limitations should be considered. Restricting inclusion to English-language publications may introduce language bias, and searching only PubMed, Scopus, and Google Scholar could have missed relevant studies in other databases. The included studies exhibited heterogeneity in animal species, extract preparation, doses, and exposure times, which may affect direct comparability. As a narrative review, this study does not provide pooled effect estimates, and publication bias cannot be fully excluded. Finally, while efforts were made to standardize data presentation, inherent variability across experimental protocols may influence the interpretation of results.

## 4. Pathways of Gastric Ulcer Pathogenesis

**Figure 1:**
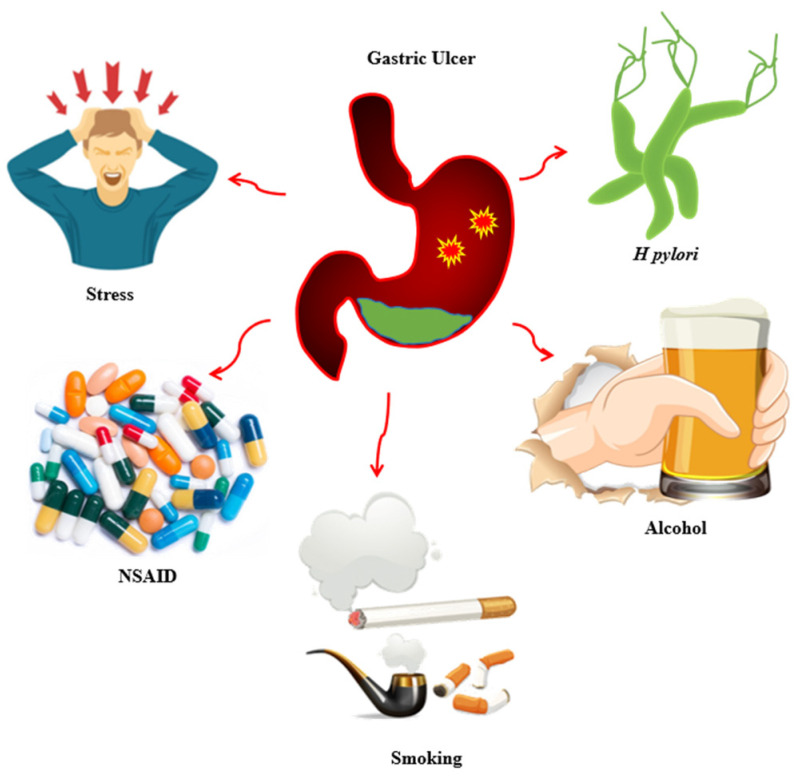
Pathways of Gastric Ulcer Induction.

Gastric ulcer disease is a multifactorial disorder resulting from an imbalance between aggressive factors and mucosal defense mechanisms. As illustrated in Figure 1, gastric mucosal injury may be triggered by microbial infection, prolonged exposure to ulcerogenic drugs, psychological and physical stress, and lifestyle-related factors, each operating through distinct pathogenic pathways. Well-established etiological factors include smoking [22], NSAID use [4], *H. pylori* infection [23], alcohol consumption [24], and stress [25]. These factors contribute to mucosal injury through mechanisms such as suppression of prostaglandin synthesis, increased gastric acid secretion, oxidative stress, inflammatory cytokine release, and neuroendocrine dysregulation [14,26,27].

Although these pathways are important for general gastric ulcer pathogenesis, ethanol-induced gastric injury involves a distinct mechanism characterized by direct mucosal damage, microvascular impairment, oxidative stress, and activation of inflammatory mediators. Therefore, this review specifically focuses on ethanol-induced gastric ulcer models to evaluate the gastroprotective potential of medicinal plant extracts within this well-established experimental framework.

## 5. Ethanol-Induced Ulcer

The ethanol-induced gastric ulcer model involves direct mucosal damage caused by excessive ethanol, resulting in hemorrhages, cellular exfoliation, inflammation, and mucosal edema [20,26,27,28]. Ethanol easily penetrates the gastric mucosa, exposing it to acidic conditions and pepsin, which damage the epithelial membrane. It also reduces gastric blood flow, disrupts vascular endothelium, and increases xanthine oxidase activity, thereby promoting oxidative stress and microvascular injury [12,29,30,31].

During ethanol metabolism, alcohol dehydrogenase converts ethanol to acetaldehyde, which xanthine oxidase can further convert to reactive oxygen species (ROS). Free radicals generated in this process decrease mucus and bicarbonate secretion, increase lipid peroxidation (LPO), and damage capillary and endothelial cells, leading to necrosis, bleeding, and epithelial erosion [3,18,32]. Vasoactive mediators such as leukotriene C4, endothelin-1, and histamine increase vascular permeability, worsening mucosal injury. Neutrophil infiltration further amplifies ROS production, damaging lipids, proteins, and other cellular components [33,34,35].

**Figure 2:**
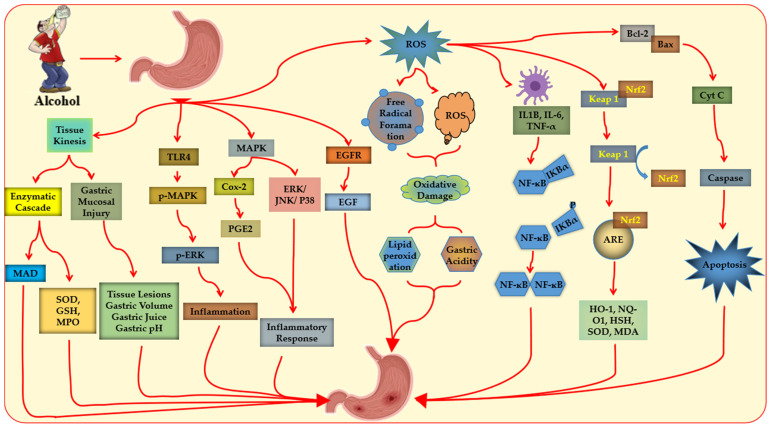
Mechanism of ethanol-induced gastric ulcer.

The molecular mechanisms of ethanol-induced gastric ulcer, involving oxidative stress, inflammation, and apoptosis, are illustrated in Figure 2. Activated neutrophils increase myeloperoxidase (MPO) activity and nuclear factor kappa B (NF-κB)-mediated production of pro-inflammatory cytokines such as TNF-α, exacerbating mucosal injury [36,37,38,39,40]. Ethanol exposure reduces levels of anti-inflammatory cytokines (e.g., IL-10) and depletes endogenous antioxidants, including total antioxidant capacity (TAC), glutathione (GSH), and glutathione peroxidase (GPx), while increasing lipid peroxidation. Additionally, ethanol lowers cytoprotective molecules such as nitric oxide (NO) and Prostaglandin E_2_ (PGE_2_), further compromising mucosal integrity [36,38,41,42,43].

## 6. Current Pharmacological Management

Currently, gastric ulcers are managed using pharmacological agents that provide mucosal protection and reduce gastric acidity. Triple therapy, consisting of two antibiotics and a proton pump inhibitor, is standard for *H. pylori*-associated peptic ulcers, whereas acid-suppressive and cytoprotective treatments are applied for ethanol-induced ulcers [44,45]. Omeprazole, a widely used proton pump inhibitor, reduces acid secretion but is associated with side effects, highlighting the need for safer therapeutic options [46,47,48].

## 7. Phytotherapy Treatment for Ethanol-Induced Gastric Ulcer

Phytotherapy—the use of medicinal plants to treat illnesses—has been employed since ancient times [47]. Plant-derived bioactive compounds, or phytochemicals, are concentrated in roots, bark, leaves, seeds, and fruits. These compounds exhibit anti-inflammatory, antioxidant, cytoprotective, and anti-secretory activities, making them valuable candidates for gastroprotective therapy [48,49,50,51,52].

In ethanol-induced gastric ulcer models, phytochemicals mitigate mucosal injury by restoring antioxidant balance, reducing ROS, modulating inflammatory pathways, and promoting tissue regeneration. Key classes include alkaloids, which regulate gastric acid secretion and provide cytoprotection [48]; Flavonoids, which accelerate ulcer healing through anti-inflammatory and antioxidant effects [49]; and essential oils and polyphenols, which protect the mucosa and exhibit anti-secretory and anti-inflammatory activities [50].

The imbalance between defensive mechanisms (mucus, PG synthesis, endothelial blood flow) and aggressive factors (acid, pepsin, ethanol, *H. pylori*) underlies ethanol-induced gastric ulcer [53]. Ethanol exacerbates mucosal damage via its hydrolytic and proteolytic effects while decreasing blood flow, increasing ROS, and elevating pro-inflammatory cytokines [8,26,53,54,55,56,57,58].

Herbal extracts demonstrate significant gastroprotective effects due to their high phytochemical content, antioxidant activity, and anti-inflammatory properties [59]. These extracts provide a complementary approach to conventional therapy, particularly in ethanol-induced gastric ulcers, where *H. pylori*-targeted quadruple therapy is not indicated. Screening for high-efficacy, low-toxicity phytochemicals is therefore essential to enhance patient outcomes and reduce reliance on drugs with adverse effects [47,60,61,62,63].

## 8. Phytotherapy-Focused Discussion

Herbal extracts are effective in treating stomach ulcers due to their high phyto-chemical content and their anti-inflammatory and antioxidant properties, as summarized in Table 1. The main treatment strategies include healing the gastric mucous membrane, managing pathogens, and reducing acid secretion [49]. Quadruple therapy (a proton pump inhibitor, bismuth, and two antibiotics) is used for *H. pylori*-related gastric ulcers, whereas ethanol-induced ulcers are managed with acid-suppressive and cytoprotective treatments [50]. The limitations of quadruple therapy, including high relapse rates, side effects, and pathogen resistance, have led to a greater need for new treatment options [51]. Modern medical research indicates that phytomedicine offers low toxicity and multiple targeting effects, making it suitable for integration with quadruple therapy in the treatment of gastric ulcers [16,52,53]. As a result, screening phytomedicine for high-efficiency and low-toxicity monomers is crucial for improving the quality of life of gastric ulcer patients and increasing the efficacy of treatment.

## 9. Integrative Mechanistic Perspective on Ethanol-Induced Anti-Ulcer Phytotherapeutics

Despite extensive phytochemical diversity, the evaluated plant-derived interventions converge into five principal gastroprotective pathways: (i) enhancement of mucus production and reinforcement of mucosal barrier integrity; (ii) antioxidant-mediated cytoprotection through attenuation of lipid peroxidation and restoration of endogenous enzymatic defenses (SOD, CAT, GSH); (iii) anti-secretory activity involving suppression of gastric acidity and inhibition of H^+^/K^+^-ATPase; (iv) anti-inflammatory modulation characterized by downregulation of TNF-α, NF-κB, COX-2, and related mediators; and (v) NO and PGE_2_-dependent cytoprotective signaling.

Across experimental models of ethanol-induced gastric injury, mucosal reinforcement and attenuation of oxidative stress emerge as the most consistently reported protective mechanisms. Restoration of endogenous antioxidant systems frequently parallels reductions in MDA, a marker of lipid peroxidation, indicating coordinated suppression of oxidative damage rather than isolated biochemical modulation. Importantly, antioxidant recovery often coincides with diminished inflammatory signaling, suggesting functional interplay between redox homeostasis and NF-κB-mediated cytokine regulation.

Anti-secretory mechanisms, although less universally documented, demonstrate mechanistic integration with prostaglandin-mediated mucosal defense and NO-dependent microcirculatory regulation. The recurrent overlap among these pathways supports a multi-target pharmacodynamic model in which phytotherapeutic agents mitigate ethanol-induced gastric damage through simultaneous reinforcement of epithelial integrity, restoration of redox balance, and attenuation of inflammatory cascades.

Collectively, this mechanistic convergence underscores a systems-level pattern of gastroprotection, emphasizing functional integration over botanical taxonomy and strengthening the translational relevance of phytotherapeutic interventions in experimental ulcer models.

## 10. Mechanistic Synthesis of Gastroprotective Effects

Although ethanol concentrations (50–100% *v*/*v*), administered volumes, and exposure times varied substantially among studies, a clear mechanistic convergence is evident. The majority of plant extracts significantly attenuated ulcer severity (↓ ulcer index and lesion area) while enhancing endogenous antioxidant systems (↑ GSH, SOD, CAT; ↓ MDA and lipid peroxidation) and suppressing key inflammatory mediators (↓ TNF-α, IL-1β, MPO). Several studies further demonstrated modulation of apoptosis-related proteins (↓ Bax, ↓ Caspase-3; ↑ Bcl-2), preservation of gastric mucus content, and stimulation of nitric oxide and prostaglandin pathways, suggesting integrated cytoprotective, antioxidant, and mucosal defensive mechanisms. Flavonoid- and tannin-rich extracts frequently exhibited dose-dependent effects, reinforcing the role of polyphenolic compounds as principal bioactive contributors [15,66].

The reproducibility of protective outcomes relative to reference drugs such as Omeprazole, Ranitidine, and Lansoprazole further validates the translational relevance of the ethanol-induced ulcer model [59,66,67,69,70,72,78,79,118,119,120,124].

**Figure 3:**
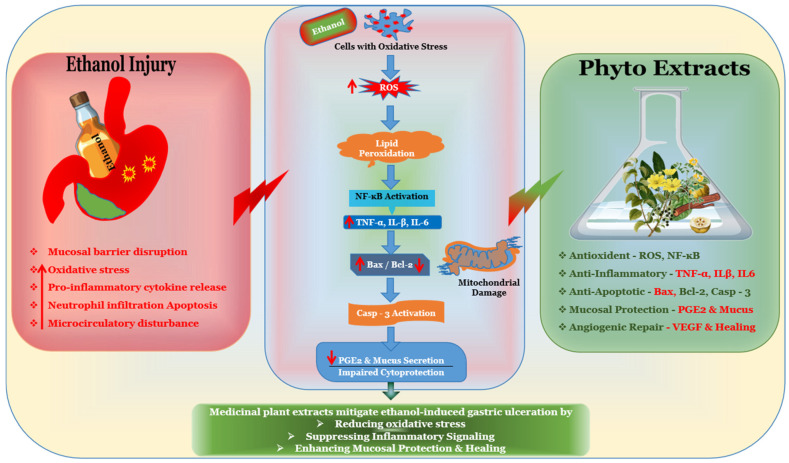
Integrated Mechanisms of Ethanol-Induced Gastric Damage and Herbal Protection.

Taken together, these findings indicate that phytochemicals exert multi-target gastroprotection primarily through coordinated antioxidant and anti-inflammatory pathways. The integrated molecular mechanisms underlying ethanol-induced gastric injury and phytochemical-mediated gastroprotection are schematically illustrated in Figure 3. Nevertheless, harmonization of dosing strategies and experimental protocols is required to strengthen quantitative comparability and translational extrapolation.

## 11. Clinical Evidence and Translational Relevance

A substantial body of experimental evidence supports the gastroprotective effects of medicinal plant extracts in ethanol-induced gastric ulcer models. However, clinical evidence in humans remains limited. Most protective effects of the plant extracts discussed in this review have been demonstrated primarily in preclinical settings, particularly rodent models of ethanol-induced gastric injury [15,34,55,66,80].

Available clinical studies and traditional usage reports suggest potential benefits of certain plants—especially those rich in flavonoids, polyphenols, and mucilaginous compounds—in reducing gastric irritation and alleviating dyspeptic symptoms. Where clinical evidence exists, we summarize the study design (e.g., clinical trial, observational study), population (healthy volunteers or patients with ulcers), intervention (extract dose and duration), and outcomes (ulcer healing, symptom improvement, or gastric protection markers). For extracts without clinical data, this is explicitly indicated [16,17,125,126].

In conclusion, while preclinical studies strongly support the gastroprotective effects of medicinal plant extracts, clinical evidence in humans is limited. Available trials and traditional reports suggest potential benefits, particularly for plants rich in flavonoids, polyphenols, and mucilaginous compounds. Further well-designed clinical studies are needed to confirm efficacy, determine optimal dosing, and assess safety before routine clinical use [17,66,80,125].

## 12. Discussion

The studies summarized in Table 1 confirm the significant gastroprotective potential of medicinal plant extracts in ethanol-induced gastric ulcer models [17,66,68,80,125]. Ethanol-induced mucosal injury results from a complex interplay of oxidative stress, inflammatory cytokine production, apoptosis, and acid-mediated epithelial damage [19,20,28,32]. Phytochemicals, particularly flavonoids, polyphenols, alkaloids, and tannins, exert coordinated protective effects by reducing ulcer severity, enhancing antioxidant defenses (↑ GSH, SOD, CAT; ↓ MDA) [37,125], suppressing inflammatory mediators (↓TNF-α, IL-1β, MPO) [33,38,44], and preserving mucosal integrity through stimulation of mucus, nitric oxide, and prostaglandin pathways [17,26,27].

Dose-dependent gastroprotection was consistently observed, with polyphenolic- and tannin-rich extracts such as *Punica granatum* [66], *Rosmarinus officinalis* [86], *Glycyrrhiza glabra* [80], and *Rhodiola rosea* [17] demonstrating robust modulation of oxidative stress, apoptosis-related proteins (Bax/Bcl-2, Caspase-3) [33,125], and inflammatory signaling [38,44]. These findings highlight the multi-mechanistic nature of phytochemical action, which extends beyond acid suppression and positions these compounds as promising candidates for complementary or preventive interventions.

Comparisons with standard anti-ulcer drugs, including Omeprazole, ranitidine, and lansoprazole, validate the ethanol-induced ulcer model and reinforce the translational relevance of the observed gastroprotective effects [45,127]. Several extracts exhibited both cytoprotective and anti-inflammatory activities, indicating that convergent mechanisms, rather than a single pathway, mediate mucosal protection [44,125].

Despite these promising preclinical outcomes, several methodological limitations warrant consideration. Heterogeneity in ethanol concentration, exposure duration, extract composition, dosage, and animal species introduces variability and limits direct quantitative comparison across studies [107,128]. Moreover, most findings are derived from rodent models, and clinical evidence in humans remains limited [17,125]. Therefore, direct extrapolation to clinical protocols should be made cautiously, and additional well-designed human studies are needed to confirm efficacy, optimal dosing, and safety.

Collectively, the evidence indicates that medicinal plant extracts act via integrated antioxidant, anti-inflammatory, cytoprotective, and anti-apoptotic mechanisms, offering multi-target gastroprotection with lower toxicity relative to conventional therapies. Standardization of extract preparation, administration, and treatment duration, along with rigorous clinical validation, is essential to translate these preclinical findings into potential therapeutic applications, particularly as adjuvant or preventive interventions for high-risk populations [17,125].

## 13. Limitations and Future Directions

Despite compelling preclinical evidence, several limitations constrain the translational applicability of phytotherapeutics for ethanol-induced gastric ulcers. Most studies are restricted to animal models with variability in species, ethanol dosing, extract composition, administration routes, and treatment durations, limiting direct clinical translation [17,18]. Sample sizes are often small, and statistical rigor is sometimes insufficient [86,129].

Key gaps remain in understanding the bioavailability, pharmacokinetics, and potential interactions of herbal compounds with conventional medications. Heterogeneity in extract composition and preparation methods further limits reproducibility. Future research should focus on standardized extracts, rigorously designed preclinical protocols, and well-controlled clinical trials to evaluate efficacy, optimal dosing, and safety. Investigating synergistic effects with conventional anti-ulcer drugs may reveal novel combination therapies and enhance translational potential [16,17,88,125].

## 14. Conclusions

Ethanol-induced gastric ulcer models consistently demonstrate the multi-target gastroprotective potential of medicinal plant extracts. Phytochemicals, particularly flavonoids, polyphenols, tannins, and alkaloids, mitigate mucosal damage through coordinated antioxidant, anti-inflammatory, cytoprotective, and anti-apoptotic mechanisms. Compared with conventional therapies, these natural compounds may exhibit lower toxicity in preclinical models and demonstrate multifaceted protective effects, making them promising candidates for further investigation as adjunctive or alternative ulcer therapies.

Although preclinical evidence is robust, however, clinical validation remains limited, and well-designed human studies are essential to confirm efficacy, determine optimal dosing, and evaluate safety. Overall, plant-derived bioactive compounds represent promising, safe, and cost-effective alternatives or adjuncts for gastric ulcer management, highlighting the translational potential of phytotherapy in future clinical practice.

## Figures and Tables

**Table 1:** Phytoextracts and bioactive constituents for gastric ulcer disease management.

Phyto Extracts	Active Constituents	Extract Dose (mg/kg or mL/kg)/bw	Ethanol Dose (kg/bw)	Animal and Body Weight (bw)	Standard Drug	Mechanistic Result	Ref.
*Davilla rugosa* stem ethanolic extract	Saponins, flavonoids, alkaloids, anthraquinones, cardiac glycosides, tannins	12.5, 335.0 and 400 mg/kg for 12 days	60% *v*/*v* (1 mL/animal), 1 h	Wistar rats (140–160 g)	Misoprostol (0.1 mg/kg)	↓ UI; ↑ mucus	[54]
*Cassia nigricans* leaves methanolic extract	Saponins, tannins, steroids, cardiac glycosides, cyanogenetic glycosides, flavonoids	250, 500 and 1000 mg/kg for 30 min	2.5 mL/kg, 4 h	Wistar rats (160–200 g)	Atropine (100 mg/kg); Yohimbine (1 mg/kg)	↓ UI	[55]
Diterpene from *Aparisthmium cordatum* bark	Diterpene	250, 100 and 500 mg/kg for 50 min	60% *v*/*v* (0.2 mL/animal), 1 h	Swiss albino mice (25–35 g)	Lansoprazole (20 mg/kg)	↓ lesions; ↑ mucus	[56]
*Turnera ulmifolia* aerial parts aqueous extract	Flavonoids, Phenolic compounds	100, 250, 500, and 1000 mg/kg for 1 h	60% *v*/*v* (0.2 mL/animal), 1 h	Swiss albino mice (30–40 g) or Wistar rats (200–220 g)	Lansoprazole (30 mg/kg)	↓ lesions	[57]
*Ocimum suave* leaves methanolic extract	Flavonoids, Tannins, Saponins, Phenolic compounds	75 and 500 mg/kg for 1 h	60% *v*/*v* (1 mL/animal), 1 h	Wistar rats (150–200 g)	Indomethacin (20 mg/kg)	↑ mucus; ↓ lesions	[58]
*Stryph-nodendron adstringens* stem bark acetone soluble fraction	Tannin	100, 400 and 800 mg/kg for 1 h	75% *v*/*v* (10 mL/kg), 1 h	Albino rats (180–200 g)	Ranitidine (50 mg/kg)	↓ gastric volume & acidity; ↑ pH	[59]
*Aegle marmelos* fruit ethanolic extract	Alkaloids, proteins, carbohydrates, tannins	50, 100, and 200 mg/kg for 1 h	100% *v*/*v* (1 mL/200 g), 1 h	Sprague Dawley rats (140–160 g)	Sulfate (0.1 mg/kg)	↓ UI; ↓ intestinal propulsion	[60]
*Opuntia ficus indica* aqueous fruit juice	Ascorbic acid, total polyphenols, flavonoids	3 mL/animal for 9 days	90% (0.5 mL/animal), 1 h	Wistar rats (180–200 g)	Sucralfate (3 mL/rat)	↑ mucus; ↓ ulceration; restored mucosal architecture	[61]
*Petroselinum crispum* ethanolic extract	Tannins, flavonoids, sterols, triterpenes	1000 and 2000 mg/kg for 30 min	80% *v*/*v* (1 mL/animal), 30 min	Wistar albino rats (150–200 g)	-	↓ UI; ↑ gastric wall mucus	[62]
*Persea americana* leaves aqueous extract	Alkaloids flavonoids, saponins, tannins	100, 200 and 400 mg/kg for 1 h	100% *v*/*v* (1 mL/kg), 1 h	Albino rats (180–250 g)	Cimetidine (100 mg/kg)	↓UI	[63]
*Manilkara hexandra* bark acetone extract	Procyanidins, flavonoids, saponins, terpenoids	500 mg kg for 1 h	50% *v*/*v* (1 mL/animal), 2 h	Wistar albino rats (200 ± 20 g)	Pantoprazole (20 mg/kg)	↓ lesions; ↓ LPO/MDA	[64]
*Protium heptaphyllum* trunk wood methanolic extract	α and β-amyrins, triterpenoid	200 and 400 mg/kg for 1 h	96% *v*/*v*, 30 min; Ethanol 60% *v*/*v* (0.2 mL/animal), 1 h	Wistar rats (150–200 g) and Swiss mice (20–25 g)	N-acetyl-l-cysteine (750 mg/kg)	↓ gastric injury (dose-related); ↓ gastric volume; ↑ NP-SH	[65]
*Terminalia pallida* fruit ethanolic extract	Steroids, flavonoids, tannins	250 and 500 mg/kg for 1 h	1 mL/200 g, 1 h	Swiss albino rats (180–200 g)	Omeprazole (10 mg/kg)	↓ ulcer incidence and severity	[66]
*Punica granatum* fruits methanolic extract	Alkaloids, flavonoids, polyphenols	250 and 500 mg/kg for 1 h	80% *v*/*v* (1 mL/animal), 4 h	Wistar rats (180–200 g)	Ranitidine (50 mg/kg)	↓ UI; ↓ LPO; ↑ SOD, CAT, GPx, GSH; ↓ neutrophil infiltration	[67]
*Ginkgo biloba* leaves ethanolic extract	Flavonoids, terpenoids	8.75, 17.5 and 26.25 mg/kg by iv for 30 min	50% *v*/*v* (1 mL/animal), 30 min	Wistar albino rats (180–200 g)	-	↑ NP-SH; ↓ MDA; ↓ JNK activation	[68]
*Cardiospermum halicacabum* whole plant ethanolic extract	Tannins, saponins, alkaloids	200, 400 and 600 mg/kg for 14 days	100% *v*/*v* (8 mL/kg), 30 min	Wistar rats (130–150 g)	Omeprazole (20 mg/kg); Ranitidine (150 mg/kg)	↓ ALP; ↑ GSH; ↓ TBARS	[69]
*Mouriri pusa* leaves dichloromethane and methanolic extract	Tannins, flavonoids, (−)-epicatechin	250, 500 or 1000 mg/kg for 50 min	99.5% *v*/*v* (1 mL/animal), 1 h; Ethanol 60% *v*/*v* (0.2 mL/animal), 1 h	Swiss mice (25–40 g) or Wistar rats (200–250 g)	Lansoprazole (30 mg/kg)	↓ UI; ↓ lesions	[70]
*Solanum nigrum* fruits methanolic extract	Glycoalkaloids, steroidal glycosides, steroidal saponins, steroidal genin, tannin, polyphenolic compounds.	200 and 400 mg/kg for 7 days	50% *v*/*v* (1 mL/kg), 1 h	Wistar albino rats (120–150 g)	Omeprazole (10 mg/kg)	↓ acidity, pepsin, gastrin, H^+^/K^+^-ATPase and ulcer size	[71]
*Falcaria vulgaris* leaves and stems ethanolic extract	Tannin, saponin	50, 100 and 150 mg/kg twice for 2 days	50% *v*/*v* (10 mL/kg), 1 h	Albino rats (200–250 g)	Ranitidine (50 mg/kg)	↓ UI; ↓ mucosal necrosis & edema; ↓ leukocyte infiltration	[72]
*Ocimum gratissimum* leaves methanolic extract	Flavonoids, tannins, saponins, carbohydrate, steroids, alkaloids, terpenes, volatile oils	200, 400 and 800 mg/kg for 1 h	96% *v*/*v* (1 mL/animal), 30 min	Wistar albino rats (180–200 g)	Cimetidine (100 mg/kg)	↓ lesions	[73]
*Foeniculum vulgare* aerial parts aqueous extracts	Flavonoids, sterols, tannins, coumarins	75, 150 and 300 mg/kg for 1 h	80% *v*/*v* (1 mL/animal), 1 h	Sprague Dawley rats (190–225 g)	Famotidine (20 mg/kg)	↓ MDA; ↑ GSH, nitrite/nitrate, vitamins; ↓ gastric injury	[34]
*Phyllanthus amarus* leaves acetone and aqueous extracts	Flavonoids, alkaloids, major lignans, polyphenols	100, 500 and 1000 mg/kg for 14 days	100% *v*/*v* (1 mL/200 g), 1 h	Albino rats (140–185 g)	Cimetidine (100 mg/kg)	↓ulcer damage; ↓ AST/ALT; ↓ CAT, SOD, GST	[74]
*Raphanus sativus* squeezed Radish juice aqueous extract	Flavonoids, anthocyanins, sulfurated constituents	2 and 4 mL per 200 g for 30 min	80% *v*/*v* (1 mL/200 g), 1 h	Wistar albino rats (150–200 g)	-	↑ mucus binding (Alcian blue); ↑ mucosal protection	[75]
*Polyalthia longifolia* leaves ethanolic extract	Alkaloids, Terpenoids	300 mg/kg for 1 h	60% *v*/*v* (0.2 mL/animal), 1 h	Swiss albino mice (24–30 g)	Sucralfate (100 mg/kg)	↓ gastric lesions	[76]
*Eugenia umbelliflora* leaves and fruits methanolic extract	Triterpenes, phenolic compound	50, 125 and 250 mg/kg for 1 h	99.5% *v*/*v* (1 mL/animal), 1 h	Balb–C mice (20–22 g)	Omeprazole (30 mg/kg)	↓ lesion	[77]
*Mangifera indica* leaves aqueous decoction	Phenolic constituents, triterpenes, flavonoids, phytosterols, polyphenols	250, 500 and 1000 mg/kg for 50 min	99.5% *v*/*v*, 1 h; 60% *v*/*v* (0.2 mL/animal), 1 h	Swiss albino mice (25–35 g) and Wistar albino rats (150–250 g)	Lansoprazole (30 mg/kg)	↓ gastric lesion severity	[78]
*Pongamia pinnata* seeds methanolic extract	Flavonoids, Furanoflavonoids, Terpenoids, Sterols, Phenolic compounds	12.5, 25 and 50 mg/kg twice a day for 5 days	90% *v*/*v* (1 mL/200 g), 1 h	Albino rats (150–200 g)	Omeprazole (2 mg/kg); Sucralfate (500 mg/kg)	↓ acid output; ↑ mucin & glycoproteins; ↑ CAT & GSH; ↓ LPO	[21]
*Cinnamomum tamala* leaves ethanolic extract	Flavonoids, tannins, alkaloids, volatile oils	50, 100 and 200 mg/kg twice a day for 5 days	1 mL/200 g, 1 h	Sprague Dawley rats (140–190 g) and Swiss albino mice (25–30 g)	Ranitidine (50 mg/kg)	↓ lesion index	[79]
*Phyllanthus niruri* leaf aqueous extract	Lignans, phyllanthin, hypophyllanthin, flavonoids, glycosides, tannins	250, 500, 750 and 1000 mg/kg for 30 min	100% *v*/*v* (5 mL/kg), 1 h	Albino Wistar rats (180–220 g)	Omeprazole (20 mg/kg)	↓ mucosal damage; ↓ edema & leukocyte infiltration	[80]
*Calamintha officinalis* leaves methanolic extract	Polyphenols, catechic tannins, terpenes	50, 100 and 200 mg/kg for 1 h	90% *v*/*v* (0.5 mL/100 g), 1 h	Wistar rats (180–200 g) and Swiss mice (20–25 g)	Sucralfate (100 mg/kg)	↓ lesion intensity; ↑ mucus integrity; preserved glandular structure	[81]
*Lantana camara* leaves methanolic extract	Alkaloids, saponins, glycosides, carbohydrates, tannins, flavonoids, steroids, triterpenoids	250 and 500 mg/kg for 10 days	90% *v*/*v* (5 mL/kg), 1 h	Wistar albino rats (150–200 g)	Famotidine (20 mg/kg)	↓ UI; ↓ LPO; ↑ GSH	[82]
*Physalis angulata* leaves ethanolic extract	Steroid, physagulins, flavonoids	250 and 500 mg/kg for 45 min	90% *v*/*v* (1 mL/200 g), 1 h	Albino Wistar rats (180–200 g)	Omeprazole (20 mg/kg)	↓ UI	[83]
*Gymnema sylvestre* leaves ethanolic extract	Oleanane, alkaloids, acidic glucosides, anthroquinones	100, 200 and 400 mg/kg for 1 h	80% *v*/*v* (1 mL/animal), 1 h	Wistar albino rats (200–220 g)	Cimetidine (50 mg/kg)	↓ UI; ↑ mucus, proteins, NP-SH; ↓ MDA	[84]
*Psidium guajava* leaves methanolic extract	Flavonoids, polyphenolic compounds	500 and 1000 mg/kg for 10 days	90% *v*/*v* (1 mL/animal), 1 h	Wistar rats (160–225 g)	Ranitidine (50 mg/kg)	↓ UI	[85]
*Rosmarinus officinalis* leaves ethanolic extract	Phenolics, flavonoids	500 and 1000 mg/kg for 12 h	70% *v*/*v* (2 mL/kg), 1 h	Wistar rats, (270–320 g)	Omeprazole (30 mg/kg)	↓ MDA, MPO; ↑ GSH/GSSG, CAT, NOX	[86]
*Tragopogon graminifolius* aerial parts hydroalcoholic extract	Flavonoids, triterpene, saponins	50, 100 and 150 mg/kg for 15 days	100% *v*/*v* (1 mL/200 g), 1 h	Wistar rats (190–230 g)	Omeprazole (10 mg/kg)	↓UI	[87]
*Rhus tripartitum* root bark methanolic extract	Phenolics, flavonoids, tannins, polysaccharide	50, 200 and 400 mg/kg for 1 h	80% *v*/*v* (0.5 mL/animal), 1 h	Wistar rats (240–260 g)	Ranitidine (50 mg/kg)	↓ gastric juice & acidity; ↑ mucus; ↓ LPO; ↑ antioxidant enzymes	[88]
*Curcuma purpurascens* rhizome n-hexane extract	Turmerone	200 and 400 mg/kg for 1 h	100% *v*/*v* (5 mL/kg), 1 h	Sprague Dawley rats (150–180 g)	Omeprazole (20 mg/kg)	↓ edema & MDA; ↑ SOD & NO	[89]
*Crassocephallum vitellinum* aerial parts ethanolic extract	Tannins, saponins, flavonoids, terpenoids	200, 400 and 800 mg/kg for 1 h	80% *v*/*v* (5 mL/kg), 4 h	Sprague Dawley rats (100–188 g)	Pantoprazole (40 mg/kg)	↓ UI	[90]
*Scutia buxifolia* stem bark ethanolic extract	Phenolic content, flavonoids, tannins	200 and 400 mg/kg for 1 h	70% *v*/*v* (0.5 mL/100 g), 1 h	Wistar rats (200–250 g)	Omeprazole (30 mg/kg)	↓ mucosal damage; ↓ MDA; ↑ CAT, SOD, NP-SH	[91]
*Mentha longifolia* plants ethanolic extract	Flavonoids, tannins, saponins	100 and 200 mg/kg for 1 h	80% *v*/*v* (1 mL/animal), 1 h	Wistar albino rats (150–200 g)	Ranitidine (20 mg/kg)	↓ UI	[92]
*Ziziphus jujuba* stem bark aqueous extract	Tannin, flavonoids, alkaloids, terpenoids	100, 200 and 400 mg/kg for 1 h	80% *v*/*v* (5 mL/kg), 2 h	Albino Wistar rats (230–270 g)	Ranitidine (150 mg/kg)	↓ UI	[93]
*Piptadeniastrum africanum* stem bark aqueous and methanolic extract	Alkaloids, flavonoids, phenols, saponins	125, 250 and 500 mg/kg for 1 h	60% *v*/*v* (1 mL/150 g), 1 h	Wistar rats (180–220 g)	Ranitidine (50 mg/kg)	↓ gastric ulceration	[94]
*Achillea biebersteinii* aerial part ethyl acetate extract	Terpenes, flavonoids, phenolic acids, sesquiterpene lactones, ionone glucosides, terpenoids	200 mg/kg for 7 days	100% *v*/*v* (0.5 mL/100 g), 1 h	Wistar albino rats (100–120 g)	Ranitidine (100 mg/kg)	↓ lesion count; ↓ acidity & volume; ↓ MDA; ↑ GSH, SOD	[95]
*Eruca sativa* leaf ethanolic extract	Flavonoids	250, 500 and 750 mg/kg for 1 h	95% *v*/*v* (5 mL/kg), 30 min	Rattus norvegicus rats (150–200 g)	Omeprazole (20 mg/kg)	↓ ulcer area; ↓ acidity; ↓ edema & infiltration	[41]
*Morus macroura* fruit ethanolic extract	Flavonoids, phenolic acids	300 mg/kg for 7 days	100% *v*/*v* (5 mL/kg), 2 h	Sprague Dawley rats (150–200 g)	Ranitidine (100 mg/kg)	↓ lesion severity; ↓ acidity; ↓ TNF-α; ↓ PGE_2_	[96]
*Osyris quadripartita* leaf methanolic extract	Flavonoids, tannins, saponins	100 and 400 mg/kg for 10 day	90% *v*/*v* (1 mL/200 g), 1 h	Wistar albino rats (160–200 g)	Sucralfate (100 mg/kg)	↓ UI; ↓ acidity; ↑ pH	[97]
*Actinodaphne sesquipedalis* leaf methanolic extract	Alkaloids, saponins, sterols and triterpenes, polyphenols, tannins	150 and 300 mg/kg for 1 h	100% *v*/*v* (5 mL/kg), 1 h	Rats (175–210 g)	Omeprazole (20 mg/kg)	↓ acidity; ↑ mucus & glycoproteins; ↑ Hsp70, GSH, NO; ↓ Bax & MDA	[98]
*Phyllanthus niruri* leaves methanolic extract	Flavonoids, alkaloids, terpenoids, lignin, polyphenols, tannins, coumarins, saponins	100, 200 and 400 mg/kg for 30 min	60% *v*/*v* (25 mL/kg), 90 min	Swiss albino rats (120–150 g)	Omeprazole (20 mg/kg)	↓ UI	[99]
*Limonia acidissima* leaves ethanolic extract	Alkaloids, flavonoids, steroids, saponins, glycosides, phenols, gum and mucilage, fixed oils and fats, resins, tannins	200 and 400 mg/kg for 1 h	100% *v*/*v* (1 mL/kg), 2 h	Albino rats (110–200 g)	Ranitidine (20 mg/kg)	↓ gastric volume & ulcer area; ↑ pH	[100]
*Cyrtocarpa procera* stem bark methanolic extract	Triterpenes, Phytosterols, Terpenoids	300 mg/kg, twice a day for 20 days	100% *v*/*v* (7 mL/kg), 1 h 30 min	CD–1 mice (20–25 g)	-	↓ ulcer area; ↑ NO & PGs-mediated protection	[101]
*Salvadora persica* root aqueous extract	Vitamin C, tannins, saponins, flavonoids	200 and 400 mg/kg for 7 days	100% *v*/*v* (5 mL/kg), 1 h	Rats (240–250 g)	-	↓ edema & necrosis; ↓ NF-α, IL-1β, iNOS; ↑ eNOS, TGF-β1	[102]
*Cuphea ignea* aerial parts ethanolic extract	Phenolics, flavonoids, tannins, alkaloids, carbohydrates, glycosides, triterpenes, unsaturated sterols	250 and 500 mg/kg for 7 days	100% *v*/*v* (1.5 mL/animal), 1 h	Sprague Dawley rats (150–200 g)	Ranitidine (30 mg/kg)	↓ UI; ↓ acidity; ↑ pH; ↑ CAT, SOD, GSH; ↓ MDA	[103]
*Myristica fragrans* seeds ethanolic extract	Alkaloids, flavonoids, tannins, phlobatannins, quinones, anthraquinones, acids, resins, terpenoids, fatty acids, volatile oils, glycosides	200 mg/kg for 14 days	90% *v*/*v* (5 mL/kg), 1 h	Wistar albino rats (150–300 g)	Sucralfate (100 mg/kg)	↓ lesion index; ↓ acidity; ↑ pH	[104]
*Erythrina speciosa* leaves methanolic extract	Alkaloids, flavonoids, saponins	25, 50 and 100 mg/kg for 1 h	100% *v*/*v* (5 mL/kg), 1 h	Sprague Dawley rats (210 ± 10 g)	Omeprazole (20 mg/kg)	↓ lesions; ↑ mucin; ↓TNF-α, NF-κB, COX-2	[105]
*Lavandula stoechas* arial parts aqueous and methanolic extract	Glycosides, alkaloids, tannins, flavonoids, saponins, carbohydrates, proteins, terpenoids	300 mg/kg for 1 h	80% *v*/*v* (1 mL/animal), 5 h	Albino rats	Omeprazole (4 mg/mL) and Ranitidine (25 mg/mL)	↓ UI; ↑ mucosal integrity; antioxidant activity	[106]
*Dissotis rotundifolia* whole plant methanol extract	C-glycosylflavones	100 and 300 mg/kg for 14 days	100% *v*/*v* (200 mg/kg), 1 h	Sprague Dawley rats (200–250 g)	Omeprazole (30 mg/kg)	↓ edema & hemorrhage; ↓ MDA; ↑ CAT & SOD	[107]
*Pachira glabra* leaves methanolic extract	Flavonoids, Phenolic acids	100, 200, or 400 mg/kg for 1 h	100% *v*/*v* (5 mL/kg), 1 h	Wistar rats (200–220 g)	Omeprazole (20 mg/kg)	↓ lesion index; ↓ COX-2, NF-κB, Bax; ↑ Bcl-2	[108]
*Ononis spinosa* leaves methanolic extract	Isoflavone glycoside, flavonoid glycoside, phenolic acid, triterpenoid compound	500 and 1000 mg/kg for 1 h 30 min	100% *v*/*v* (5 mL/kg), 1 h	Wistar rats (220–240 g)	Esomeprazole (40 mg/kg)	↓ UI; ↓ edema & hemorrhage; ↑ GSH	[109]
*Dicliptera bupleuroides* methanolic extract	Phenols, flavonoids, ascorbic acid, lipids, starch, glycosides	500 mg/kg	100% *v*/*v* (1 mL/animal)	Wistar albino rats (250 ± 30 g)	Ranitidine (50 mg/kg), Omeprazole (20 mg/kg), and Sucralfate (100 mg/kg)	↓ gastric volume & acidity; ↑ pH	[110]
*Sarcandra glabra* aqueous extract	Isofraxidin, rosmarinic, caffeic acid	10,000 mg/kg daily for 7 days	100% *v*/*v* (5 mL/kg), 1 h	Sprague Dawley (180–200 g)	-	↓ mucosal damage; ↓ MDA, TNF-α, IL-6; ↑ PGE_2_, NO, SOD	[111]
*Fumaria officinalis* aerial parts ethanolic extract	Carbohydrates, saponins, flavonoids, phytosterols, tannins, phenolic	200 and 400 mg/kg for 21 days	100% *v*/*v* (5 mL/kg), 1 h	Wistar Albino rats (200–250 g)	Esomeprazole (20 mg/kg)	↓ UI & acidity; ↓ edema & inflammation	[112]
*Carica papaya* leaves aqueous and methanolic extract	Flavonoids, tannins, alkaloids, terpenoids, cardiac glycosides, reducing sugar, saponins,	300 and 600 mg/kg for 14 days	95% *v*/*v* (1 mL/animal), 2 h	Wistar rats (120–150 g)	Omeprazole (25 mg/kg)	↓ gastric lesions	[113]
*Ocimum gratissimum* leaves methanolic, n-hexane and ethyl acetate extracts	Alkaloids, flavonoids, tannins, terpenoids, carbohydrates, steroids, saponin	200 and 400 mg/kg for 14 days	100% *v*/*v* (1 mL/animal), 1 h	Albino rats (150–180 g)	Omeprazole (20 mg/kg)	↓ ulcer	[114]
*Heterotis rotundifolia* stem n-hexane, ethyl acetate and methanolic extract	Terpenes, flavonoids, tannins, alkaloid, glycosides	300, 600 and 900 mg/kg for 1 h	2.5 mL/kg, 4 h	Swiss albino rats (145–170 g)	Propranolol (40 mg/kg)	↓ UI	[115]
*Parapholis incurvaon* aerial parts hydroethanolic extract	Phenolic Compounds, Flavonoids	400 mg/kg for 2 h	100% *v*/*v* (1 mL/100 g), 1 h	albino rats (150–200 g)	Ranitidine (50 mg/kg)	↓ MDA; ↑ GSH; preserved mucosal epithelium	[116]
*Solanum melongena* peels alcoholic extract	Phenolic Acids, Flavonoids	150, 250, and 500 mg/kg for 7 days	100% *v*/*v* (0.5 mL/100 g), 1 h	Wistar albino rats (100–120 g)	Metronidazole (20 mg/kg), Famotidine	↓ acidity & lesion number; ↑ pH; ↑ SOD; ↓ MDA	[117]
*Rhodiola rosea* root methanolic extract	Phenolic acids, tannins, rosavins, salidroside	300 and 600 mg/kg for 7 days	100% *v*/*v* (2 mL/animal), 1 h	Wistar rats (200–220 g)	Omeprazole (10 mg/kg)	↓ UI & acidity; ↑ PGE_2_, NO, SOD, CAT, GSH; ↓ TNF-α, ILs	[118]
Chinese yam aqueous extract	Organic acids, amino acids, carbohydrates	310, 630, and 3140 mg/kg for 14 days	100% *v*/*v* (10 mL/kg), 2 h	ICR mice	Omeprazole (20 mg/kg)	↑ SOD, GPx, CAT; ↓ MDA, TNF-α; modulated Bcl-2/Bax	[119]
*Papaver decaisnei* leaves methanolic extract	Phenolics, flavonoids, terpenoids	200 and 400 mg/kg for 14 days	100% *v*/*v* (5 mL/kg), 1 h	Sprague Dawley rats (180–210 g)	Omeprazole (20 mg/kg)	↓ lesion area; ↑ mucus; ↑ HSP70; ↓ TNF-α, IL-6	[120]
*Cordia africana* roots ethyl acetate fraction	Phenolic, flavonoid	200 and 400 mg/kg for 1 h	100% *v*/*v* (1 mL/animal), 1 h	Wistar albino rats, (140–150 g)	Pantoprazole (10 mg/kg)	↓ ulcer severity; ↓ MDA, TNF-α, NF-κB; ↑ GSH, PGE_2_	[121]
*Talium triangulare* leaves aqueous extract	Flavonoid, Phenolic acids, Carotenoids, Alkaloids, tannins, saponins, Benzoic acid derivatives, hydroxycinnamates	200 mg/kg for 14 days	100% *v*/*v* (1 mL/animal), 1 h	Albino Wistar rats	Omeprazole (20 mg/kg)	↓ UI; ↓ MDA, MPO, TNF-α; ↑ GSH	[15]
*Pancratium maritimum* ethanolic extract	Amino acids, organic acids, phenolic acids, alkaloids, flavonoids, fatty acids	25, 50, and 100 mg/kg for 7 days	96% *v*/*v* (1 mL/kg), 1 h	Wistar rats (200–250 g)	Omeprazole (20 mg/kg)	↓MDA, TNF-α, NF-κB, Casp-3	[122]
*Glycyrrhiza glabra* root aqueous extract	Tannin, carbohydrates, phenols, resins, flavonoids, saponins, alkaloids, terpenes, steroids	250–500 mg/kg for 10 days	99.5% *v*/*v* (1 mL/kg), 1 h	Rats (200–300 g)	Omeprazole (0.6 mg/kg)	↓ UI; ↓ TNF-α; modulated pH	[123]

Ethanol concentrations, volumes, and exposure durations were applied as reported in the original studies, reflecting variations in experimental design. Extract doses are expressed in mg/kg body weight unless otherwise specified. Ethanol concentrations are presented as % (*v*/*v*), with units standardized where necessary. ↑ indicates increase; ↓ indicates decrease; Abbreviations: UI, ulcer index; MDA, malondialdehyde; GSH, reduced glutathione; SOD, superoxide dismutase; CAT, catalase; MPO, myeloperoxidase; LPO, lipid peroxidation; NO, nitric oxide; PGE_2_, prostaglandin E_2_; TNF-α, tumor necrosis factor alpha; IL, interleukin; NP-SH, non-protein sulfhydryls.

## Data Availability

This manuscript is a review article and does not report new experimental data. All information and data discussed are sourced from previously published studies, which are publicly available through the cited references.
